# Risk Stratification Using High-Sensitivity Cardiac Troponin T in Patients With Suspected Acute Coronary Syndrome

**DOI:** 10.1016/j.jacc.2019.12.036

**Published:** 2020-03-03

**Authors:** Andrew R. Chapman, Dennis Sandeman, Amy V. Ferry, Stacey Stewart, Fiona E. Strachan, Ryan Wereski, Anda Bularga, Atul Anand, Anoop S.V. Shah, Nicholas L. Mills

High-sensitivity cardiac troponin assays allow the early recognition of myocardial injury, and they have facilitated the development of early rule-out pathways to identify patients who do not have acute myocardial infarction. Although international guidelines recommend using the sex-specific 99th centile from a healthy reference population as the diagnostic threshold for myocardial infarction, it is increasingly evident that the use of lower thresholds to risk-stratify patients and rule-out myocardial infarction at presentation is safer and more effective.

We previously defined the optimal risk stratification threshold as the highest troponin concentration that gave a negative predictive value (NPV) for myocardial infarction or cardiac death at 30 days of 99.5%, to maximize effectiveness while maintaining safety (1). This goal was achieved by using a high-sensitivity cardiac troponin I assay at a concentration <5 ng/l, which identified two-thirds of patients as low risk at presentation and misclassified <1 in 200 patients. The performance of this risk stratification threshold has now been validated for both cardiac troponin I and T (2). We developed a simple early rule-out pathway, which incorporates separate risk stratification and diagnostic thresholds, and recognizes that small changes within the reference range may be important in those with intermediate troponin concentrations (3). In a prospective, stepped wedge, cluster randomized controlled trial, we recently showed that the introduction of this early rule-out pathway into clinical practice was both safe and effective (4). Whether this pathway performs equally well using the U.S. Food and Drug Administration–approved Roche Elecsys (Roche Diagnostics, Basel, Switzerland) high-sensitive cardiac troponin T assay is uncertain.

We recruited patients with suspected acute coronary syndrome from the emergency department of the Royal Infirmary of Edinburgh, a tertiary care hospital in Scotland, between June 1, 2013, and March 31, 2017, into a substudy of the High-STEACS (High-Sensitivity Troponin in the Evaluation of Patients With Acute Coronary Syndrome) trial (3). We did not enroll patients with ST-segment elevation myocardial infarction, those unable to provide consent, or those from outside our region to ensure complete follow-up. Blood samples were obtained at presentation and at 3 and 6 to 12 h. Samples were centrifuged and stored at –80^o^C for batch processing. This clinical trial was registered (NCT01852123) and approved by the research ethics committee; patients provided written informed consent. The final diagnosis was adjudicated by 2 cardiologists, with consensus from a third where required.

**Figure 1:**
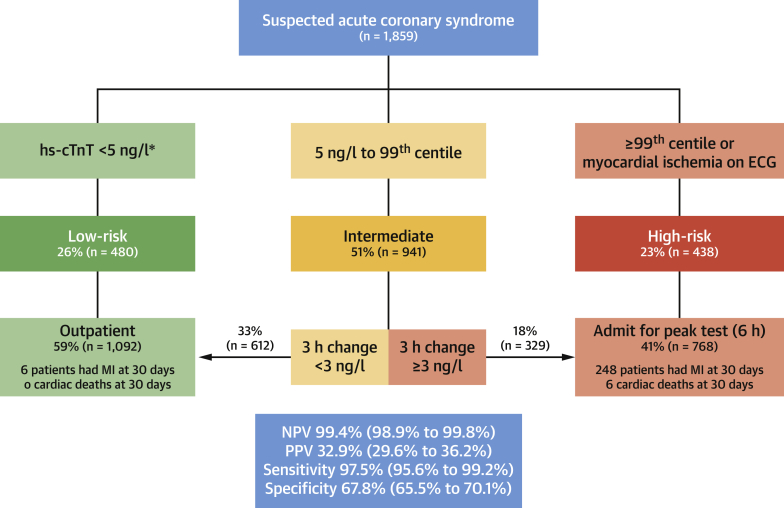
Performance of the High-STEACS Early Rule-Out Pathway for Cardiac Troponin T Myocardial infarction (MI) is ruled out when cardiac troponin concentrations are <5 ng/l at presentation. *Patients with concentrations ≥5 ng/l and those who present within 2 h of symptom onset are retested 3 h after presentation, and MI ruled out if concentrations are unchanged (delta <3 ng/l) and below the 99th centile (14 ng/l). ECG = electrocardiography; High-STEACS = High-Sensitivity Troponin in the Evaluation of Patients With Acute Coronary Syndrome; hs-cTnT = high-sensitivity cardiac troponin T; NPV = negative predictive value; PPV = positive predictive value.

We evaluated the performance of our rule-out pathway (Figure 1) applied by using the Roche Elecsys fifth-generation cardiac troponin T assay (limit of detection: 5 ng/l; 99th centile: 14 ng/l). In recognition of the U.S. Food and Drug Administration requirement not to report at a concentration of 5 ng/l, we performed a sensitivity analysis using a concentration of 6 ng/l at presentation. The primary outcome was type 1 myocardial infarction or cardiac death within 30 days.

We enrolled 1,951 patients with suspected acute coronary syndrome, of whom 1,859 had a cardiac troponin T result available at presentation. Myocardial injury was detected in 27.4% (509 of 1,859) of patients, with an adjudicated diagnosis of type 1 or type 2 myocardial infarction in 254 (13.7%) and 66 patients (3.6%), acute or chronic myocardial injury in 187 patients (10.1%), and 6 deaths from a cardiac cause at 30 days. The pathway identified 58.7% (1,092 of 1,859) of patients as low risk, with 6 missed events (5 index and 1 type 1 myocardial infarction at 30 days), for an NPV of 99.4% (95% confidence interval [CI]: 98.9% to 99.8%) and sensitivity of 97.5% (95% CI: 95.6% to 99.2%). This outcome compared favorably to the European Society of Cardiology 3-h pathway (5), which identified 64.2% (1,193 of 1,859) of patients as low risk, with 47 missed events (NPV: 96.0% [95% CI: 94.8% to 97.1%]; sensitivity: 81.7% [95% CI: 76.9% to 86.3%]). In the modified pathway using 6 ng/l at presentation, a similar performance was observed, with 59.2% (1,101 of 1,859) of patients identified as low risk, for an NPV of 99.3% (95% CI: 98.8% to 99.7%) and sensitivity of 97.1% (95% CI: 95.0% to 99.0%).

The High-STEACS early rule-out pathway seems both safe and effective when using a high-sensitivity cardiac troponin T assay, and it provides clinicians with a simple approach to triage patients with suspected acute coronary syndrome. Although our data are observational, they are consistent with the findings of our randomized controlled trial, and the pathway seems safer than guideline-recommended approaches using the 99th centile.
